# Space-time spatial channel modulation with multiple constellations

**DOI:** 10.1038/s41598-025-21284-z

**Published:** 2025-10-24

**Authors:** Yanfei Zhu, Fuchun Huang, Zhili Zhou, Zhi Yang

**Affiliations:** 1 Hubei Normal University, Huangshi, 435002 China; 2 Sun Yat-sen University, Guangzho, 510275 China; 3 Guangzhou College of Commerce, Guangzhou, 511363 China; 4 College of Electrical and Electronic Engineering, Wenzhou University, Wenzhou, 325035 China; 5 Tencent Technology Company Limited, Shenzhen, 518000 China

**Keywords:** Extension of antenna index vectors, three dimension (TD), AI set, squared minimum Euclidean distance (MED), Energy science and technology, Engineering

## Abstract

In this paper, a new schematic of spatial channel modulation aided space-time block code (STBC-SCM), which is to exploit more additional information with both the hybrid constellations group indexes (CGI) and the channel state indexes, is proposed to achieve the higher spectral efficiency and the improvement of the bit error rate (BER) performance. Specifically, four groups of mixing two signal constellations are designed for the selection of two CGI bits and encoded into one STBC codeword. Then, it is modulated on two active transmit antennas (TAs) using the antenna index (AI) bits. Furthermore, its two symbols on each active TAs are transmitted over the specified channel states with two independent parts of channel index (CI) bits, respectively. Finally, the minimum Euclidean distances with each time-slot, the detection complexity and the bit error probability are analyzed. Simulation results are shown that the performance improvement for the STBC-SCM is obviously achieved over other traditional schemes such as space-time channel modulation (STCM) and quadrature spatial modulation aided media based modulation (QSM-MBM).

## Introduction

Media based modulation (MBM)^1,2^, which is also referred to as channel modulation (CM) in Ref.^3^, has attracted remarkable attention of researchers on the exploitation of the channel index (CI) domain from the channel states. In the CM system, that each transmit antenna (TA) is equipped with $n_{\textrm{rf}}$ radio frequency (RF) mirrors that can create $2^{n_{\textrm{rf}}}$ mirror activation patterns (MAPs) with ON/OFF status of those mirrors, is the extension of the TA spatial domain. Furthermore, generalized spatial modulation (GSM)^4^, which achieves the multiplex gain, is combined with the MBM technique to form the GSM-MBM framework for improving the spectral efficiency (SE) and communication quality. With the development of the variants of the multiple-input multiple-output (MIMO) with index modulation, the quadrature spatial modulation (QSM)^5^ has been proposed to extend the spatial domain for the exploitation of the antenna index (AI) bits, i.e., developing the in-phase and quadrature dimensions. Then, with the in-phase and quadrature dimensions and, the QSM is directly integrated with the MBM system, forming the quadrature spatial MBM (QSMBM)^6^ for being capable of achieving the large SE as compared with the QSM. Furthermore, with the aid of a reserved TA, quadrature channel modulation (QCM)^7^ is proposed to avoid the overlap of the specified transmit channels for the transmission of two components of mapped signal symbol, and then to further improve the SE and reliability of communication. Recently, utilizing the variability of active TAs, the variability of active antennas aided channel modulation (VA-CM)^8^ is proposed to further lower the bit error rate(BER).

Based on the above-mentioned works, that the MBM technique is applied to the index modulation systems, greatly improves the SE and the reliability of transmission link. However, the MBM technique exploits the channel index domains without the transmit diversity gain. With the design idea of achieving the diversity gain in the GSM system^9^, this work of single-symbol GSM-MBM (S-GSM-MBM)^10^ is proposed to convey one mapped symbol on not only multiple specified active TAs but also multiple specified channel states. However, the transmit diversity gain is limited. In 1998, the concept of space-time block code (STBC)^11^ was emerged, utilizing the space and time domains to achieve two orders of transmit diversity. Then, space-time block coded (STBC) spatial modulation (STBC-SM)^12^ has been proposed to introduce the transmit diversity to the index modulation systems. In order to improve the error performance by developing the transmit diversity, the STBC technique is combined with the QSM system for forming the space-time QAM (ST-QSM) system reported in Ref.^13^. To achieve the higher transmit diversity, Euclidean geometry based space-time block coded spatial modulation (EG-STBC-SM)^14^ is proposed to improve the error performance.

In recent years, to improve the spectral efficiency, the application of the MBM technique to the STBC-SM is proposed in Reference^15^ for achieving the transmit diversity in the MBM-based index modulation systems. Furthermore, a lot of works on the combinations of the STBC with the MBM-based index modulation have been done in the references^3,16^. In the space-time channel modulation (STCM) system^3^, two mapped symbols are conveyed on not only two specified TAs and but also two specified channel states. In the space-time MBM system^16^, the transmit diversity gain is achieved with a single RF chain. In Ref.^17^, the proposed coordinate interleaved orthogonal design with MBM (CIOD-MBM) uses coordinate-interleaved orthogonal design to attain the second-order diversity. However, the CIOD-MBM’s diagonal structure fails to exploit channel states in the anti-diagonal direction of the signal matrix, constraining the SE. To address this, by deconstructing the weighted Alamouti code (WA) into real/imaginary components of its diagonal/anti-diagonal elements and deploying a dual-mode transmission mechanism, weighted Alamouti MBM^18^ (WA-MBM) activates all spatial resources across two antenna groups and time slots. This approach preserves full second-order diversity while significantly enhancing the SE. In Ref.^19^, labelling diversity is applied to the STBC-SM and media-based space-time block coded spatial modulation (MBSTBC-SM) for improving the error performance. With the limitation of the differential systems, the differential space time media-based modulation (DSTMBM) systems is proposed^20,21^ to improve the SE. Furthermore, utilizing the in-phase and quadrature dimensions, QSM aided MBM (QSM-MBM)^22^ is designed with the dispersion matrixes carrying the matrix index information to not only enhance the transmit diversity but also improve the SE with multiple indexes of the TAs, channel states and dispersion matrixes. In

However, the existing STBC-based MBM schemes (e.g., STCM, STBC-MBM) use the same CI information to select channel states for both symbols/time-slots of a single STBC codeword. This fails to exploit the potential for transmitting additional independent CI bits per symbol/time-slot, limiting the SE. Furthermore, prior designs typically draw both symbols for the STBC codeword from the same QAM constellation. This homogeneity constrains the achievable minimum Euclidean distance (MED) between signal vectors, negatively impacting error performance. Although recent variants like the WA-MBM^18^ and DSTMBM^20,21^ systems have attempted to enhance the diversity or the SE, they still suffer from static CI allocation across time slots and homogeneous constellation limitations. These observations highlight two critical limitations inherent in prior STBC-based MBM approaches: (i) Static CI allocation across STBC time slots wastes channel state resources, and (ii) Homogeneous constellations (e.g., QAM-only) restrict the MED gains.

**Figure 1:**
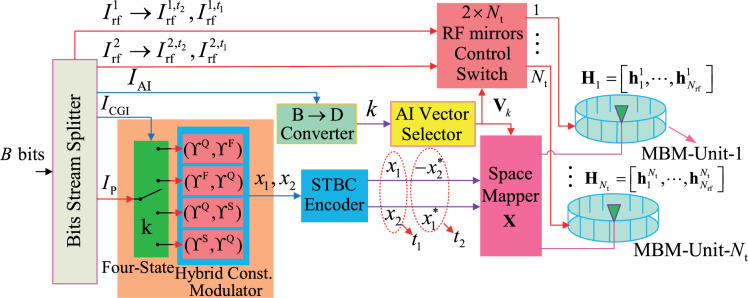
The STBC-SCM system with $N_{\textrm{t}}$ TAs whose each is equipped with $n_{\textrm{rf}}$ RF mirrors.

To overcome the above limitations, motivated by the design idea of both QSM-MBM and the hybrid constellation groups^23^, we propose a novel framework that space-time block coded spatial channel modulation (STBC-SCM) with multiple index domains such as the constellations group index (CGI), antenna index (AI) and CI domains, which leverages hybrid constellations for the CGI bits and per-symbol CI bits to enhance both the SE and MED. The objective of the proposed STBC-SCM system is to not only achieve the transmit diversity by space-time domain but also improve the SE by exploiting the CGI and CI domains. The main contributions of the proposed STBC-SCM system are summarized as below:

1. Through exploiting the CGI domain, four groups of hybrid constellations for the CGI information are designed for the STBC-SCM system. Furthermore, two symbols on two active TAs are transmitted over two specified channel states using two subparts of CI bits at each time slot. Thus, the transmit framework of the proposed STBC-SCM system can be designed in Fig. 1.
2. Then, to achieve the high SE, according to the STBC codeword, four symbols of the STBC codeword on two specified active TAs using the AI bits are modulated independently on the specified channel states with four parts of CI bits.
3. The squared MED at each time slot, the detection complexity and the average bit error probability (BEP) are analyzed. Simulation results with the maximum likelihood (ML) detector demonstrate that the STBC-SCM lowers the BER as compared with the traditional schemes such as ST-QSM, QSM-MBM and STCM.

The organization of this letter is as below. The Proposed STBC-SCM is introduced in Sect.  2. Then, Section 3 presents the performance analysis and Sect.  4 provides the BER comparisons between the STBC-SCM and different traditional schemes through simulation results. Finally, our conclusions are given in Sect.  5.

$\textbf{Notation}:$ The notations for various parameters and acronyms used throughout the paper are listed as $N_{\textrm{t}}$ is the number of transmit antennas, $\{\cdot \}^*$ is the conjugate operation, $\left\lfloor {. } \right\rfloor$ is the floor operation, $\big \Vert \cdot \big \Vert ^2$ is the Frobenius norm, $E_{\textrm{ av}}$ is the average energy per each MBM vector, $\min \{\cdot \}$ is the selecting the minimum value operation, $C_{x }^y$ is the combination operation of choosing *y* out of *x* elements, $d_{_{{\textbf { X}},\min } }^2$ is the squared minimum Euclidean distance between the MBM vectors.

## The proposed STBC-SCM

### Design of the transmitter

The transmitter of the proposed STBC-SCM system addresses critical limitations of prior STBC-based MBM schemes through three key innovations: (i) Hybrid CGI Domain: Four constellation groups: ($\Upsilon ^\textrm{Q},\Upsilon ^\textrm{F} )$, or $(\Upsilon ^\textrm{F},\Upsilon ^\textrm{Q} )$, or $(\Upsilon ^\textrm{Q},\Upsilon ^\textrm{S} )$, or $(\Upsilon ^\textrm{S},\Upsilon ^\textrm{Q} )$) are designed for the selection of CGI bits, optimizing MED while enabling additional information embedding (see in Sec. III); (ii) Dynamic CI Allocation: Independent CI bits $I^{1}_{\textrm{rf}} = \{I^{1,t_1}_{r}, I^{1,t_2}_{\textrm{rf}}\}$ and $I^{2}_{\textrm{rf}} = \{I^{2,t_1}_{\textrm{rf}}, I^{2,t_2}_{\textrm{rf}}\}$ allocate distinct channel states per symbol/time-slot, eliminating static CI reuse and increasing SE by $2n_{\text {rf}}$ bits (Eq. 4); (iii) Joint Indexing: Integration of CGI, AI ($I_{\text {AI}}$), and CI domains maximizes spatial-temporal resources without compromising diversity gain.

Specifically, as illustrated in Fig. 1 with $N_{\text {t}}$ TAs (each equipped with $n_{\textrm{rf}}$ RF mirrors), two mapped signal CPs (i.e., $x_1, x_2$) from two selected constellations (e.g., $(\Upsilon ^\textrm{Q} ,\Upsilon ^\textrm{F} )$, or $(\Upsilon ^\textrm{F},\Upsilon ^\textrm{Q} )$, or $(\Upsilon ^\textrm{Q},\Upsilon ^\textrm{S} )$, or $(\Upsilon ^\textrm{S},\Upsilon ^\textrm{Q} )$) with the CGI bits ($I_{\mathrm{{CGI}}}$) are encoded into a STBC codeword: $$\left[ {\begin{array}{*{20}c} {x_1 } & { - x_2^* } \\ {x_2 } & {x_1^* } \\ \end{array}} \right]$$, which will be introduced in the following. Note that, $\Upsilon ^\textrm{Q}, \Upsilon ^\textrm{F}, \Upsilon ^\textrm{S}$ represent the QAM, the first and second secondary QAM (FSQAM and SSQAM) constellations, respectively, e.g., 4-QAM: $\left\{ { \pm 1 \pm j} \right\}$, 4-FSQAM: $\left\{ { \pm 2, \pm 2j} \right\}$, 4-SSQAM: $\left\{ { \pm 2 \pm 2j} \right\}$.

More specifically, the input data bits of *B*, through the Bits Stream Splitter, are partitioned into five parts: $I_{\textrm{CGI}}$ being used to specify one out of four constellation groups, i.e., {$(\Upsilon ^\textrm{Q},\Upsilon ^\textrm{F} )$, $(\Upsilon ^\textrm{F},\Upsilon ^\textrm{Q} )$, $(\Upsilon ^\textrm{Q},\Upsilon ^\textrm{S} )$, $(\Upsilon ^\textrm{S},\Upsilon ^\textrm{Q} )$}, $I_{\textrm{P}}$ that is mapped into two signal CPs: $x_1, x_2$ from the selected constellation group, $I_{\textrm{AI}}$ for the selection of the AI vectors activating two active TAs, both $I^1_{\textrm{rf}}$ and $I^2_{\textrm{rf}}$ that are for the selection of the CI vectors specifying the channel states. Specifically, to exploit the CGI bits while maintaining the merit of the squared MED between the MBM vectors, three types of signal constellations ($\Upsilon ^\textrm{Q},\Upsilon ^\textrm{F}, \Upsilon ^\textrm{S}$) are used to construct four constellation groups such as $\{(\Upsilon ^\textrm{Q} ,\Upsilon ^\textrm{F} ),(\Upsilon ^\textrm{F} ,\Upsilon ^\textrm{Q} ),(\Upsilon ^\textrm{Q} ,\Upsilon ^\textrm{S} ),(\Upsilon ^\textrm{S} ,\Upsilon ^\textrm{Q} )\}$ in Hybrid Constellation Modulator for the selection of the $I_{\textrm{CGI}}$ bits. Then, with the aid of Four-State Switch controlled by the part of $I_{\textrm{CGI}}$, the part of $I_{\textrm{P}}$, containing $\log _2 (M_1 M_2)$ bits, is fed into the specified constellation group and then mapped into two signal CPs $x_1, x_2$ from the $M_1$-QAM constellation $\Upsilon ^\textrm{Q}$ and the $M_2$-FSQAM/SSQAM constellation $\Upsilon ^\textrm{F}$/$\Upsilon ^\textrm{S}$, respectively. Then, through the STBC Encoder, two signal CPs $x_1, x_2$ are encoded into one STBC codeword $$\left[ {\begin{array}{*{20}c} {x_1 } & { - x_2^* } \\ {x_2 } & {x_1^* } \\ \end{array}} \right]$$.

For part of $I_{\textrm{AI}}$, containing $N_{\textrm{AI}} = 2^{\left\lfloor {\log _2 C_{N_{\textrm{t}} }^\textrm{2} } \right\rfloor }$ bits, it is used to select an AI vector $\textbf{V}_k$ from the AI vector set $\Gamma = \left\{ {\textbf{V}_1 , \cdots ,\textbf{V}_{N_{\textrm{AI}} } } \right\}$ that has $N_{\textrm{AI}}$ vectors placed in the AI Vector Selector, whose each vector contains two non-zero elements with ”1”, where *k* is obtained by converting the $I_{\textrm{AI}}$ into a decimal number with the aid of B$\rightarrow$D Converter and $C_{y }^{x}$ is the combination of operation, selecting *x* out of *y* elements. Then, the resulted STBC codeword is modulated by the selected AI vector $\textbf{V}_k$ on the $\tau _{k,1}$ and $\tau _{k,2}$ active TAs. Thus, for two time-slots, the signal space vector (SV) $\textbf{X}$ can be generated and given by

1 $$\begin{aligned} \begin{array}{l} \textbf{X} = [\begin{array}{*{20}c} {\textbf{X}_{t_1 } } & {\textbf{X}_{t_2 } } \\ \end{array}] = [\begin{array}{*{20}c} {x_1 \cdot \textbf{e}_{\tau _{k,1} } + x_2 \cdot \textbf{e}_{\tau _{k,2} } } & { - x_2^* \cdot \textbf{e}_{\tau _{k,1} } + x_1^* \cdot \textbf{e}_{\tau _{k,2} } } \\ \end{array}] \\ \end{array}, \end{aligned}$$

where $\textbf{e}_{\tau _{k,1}}, \textbf{e}_{\tau _{k,2} } \in C^{N_{\textrm{t}}\times 1}$ are the unit vectors having one ”1” element with the $\tau _{k,1}, \tau _{k,2}$-th row positions of the AI vector $\textbf{V}_k$ corresponding to the $\tau _{k,1}$, $\tau _{k,2}$-th active TAs, respectively.

Due to that each TA is quipped with $n_{\textrm{rf}}$ RF mirrors, whose each mirror is turned ON or OFF status, $N_{\textrm{rf}}=2 ^ {{ n}_{\textrm{rf }}}$ MAPs for $2 ^ {{ n}_{\textrm{rf }}}$ channel states are generated. To achieve more CI information, four symbols (i.e., ${x_1 }, { - x_2^* }, {x_2 }, {x_1^* }$) on the $\tau _{k,1}$, $\tau _{k,2}$-th active TAs are specified by four independent CI bits to different channel states, respectively. Specifically, the two parts of $I^1_{\textrm{rf}}$ and $I^2_{\textrm{rf}}$ are respectively partitioned into two subparts of $I^{1, t_1}_{\textrm{rf}}$ and $I^{1, t_2}_{\textrm{rf}}$, $I^{2, t_1}_{\textrm{rf}}$ and $I^{2, t_2}_{\textrm{rf}}$ for two time-slots, each with $n_{\textrm{rf}}$ CI bits. At the $t_1/t_2$-th time-slot, with the aid of the AI vector $\textbf{V}^{t_1}_k$, the $2\times N_{\textrm{t}}$ RF mirrors Control Switch feeds both $I^{1, t_1}_{\textrm{rf}}$/$I^{1, t_2}_{\textrm{rf}}$ and $I^{2, t_1}_{\textrm{rf}}$/$I^{2, t_2}_{\textrm{rf}}$ into the MBM-Unit-$\tau _{k,1}$ and the MBM-Unit-$\tau _{k,2}$, and then two channel states: $\textbf{h}^{\tau _{k,1}}_{\alpha _{1}}$/$\textbf{h}^{\tau _{k,1}}_{\beta _1}$ and $\textbf{h}^{\tau _{k,2}}_{\alpha _{2}}$/$\textbf{h}^{\tau _{k,2}}_{\beta _2}$ are specified with the decimal representation $\alpha _{1}/\beta _1$, $\alpha _{2}/\beta _2$ of $I^{1, t_1}_{\textrm{rf}}$/$I^{1, t_2}_{\textrm{rf}}$, $I^{2, t_1}_{\textrm{rf}}$/$I^{2, t_2}_{\textrm{rf}}$, respectively, i.e., the two symbols $x_1/-x^*_2,~ x_2/x^*_1$ are respectively conveyed over the $\alpha _{1}/\beta _1$- and $\alpha _{2}/\beta _2$-th channel states.

Thus, before the transmission over the specified channel states, the MBM vectors for two time-slots can be given by

2 $$\begin{aligned} { \tilde{\textbf{X}}}_{\textrm{M}} = [\begin{array}{*{20}c} {{ \tilde{\textbf{X}}}_{t_1 } } & {{ \tilde{\textbf{X}}}_{t_2 } } \\ \end{array}], \end{aligned}$$

where ${ \tilde{\textbf{X}}}_{t_1 }$ and ${ \tilde{\textbf{X}}}_{t_2 }$ are given by Eq. (3).

3 $$\begin{aligned} \begin{array}{l} { \tilde{\textbf{X}}}_{t_1 } = [\underbrace{\textbf{0}_{1 \times N_{\textrm{rf}} } }_{1\mathrm{st~ TA}}, \cdots ,\underbrace{(x_1 \cdot \textbf{e}_{\alpha _1 } )^T }_{\tau _{k,1} \mathrm{th ~TA}}, \cdots ,\underbrace{\textbf{0}_{1 \times N_{\textrm{rf}} } }_{(\tau _{k,2} - 1)\mathrm{th ~TA}},\underbrace{(x_2 \cdot \textbf{e}_{\alpha _2 } )^T }_{\tau _{k,2} \mathrm{th ~TA}}, \cdots ,\underbrace{\textbf{0}_{1 \times N_{\textrm{rf}} } }_{N_{\textrm{t}} \textrm{th TA}}]^T \\ { \tilde{\textbf{X}}}_{t_2 } = [\underbrace{\textbf{0}_{1 \times N_{\textrm{rf}} } }_{1\mathrm{st ~TA}}, \cdots ,\underbrace{( - x_2^* \cdot \textbf{e}_{\beta _1 } )^T }_{\tau _{k,1} \mathrm{th ~TA}}, \cdots ,\underbrace{\textbf{0}_{1 \times N_{\textrm{rf}} } }_{(\tau _{k,2} - 1)\mathrm{th ~TA}},\underbrace{(x_1^* \cdot \textbf{e}_{\beta _2 } )^T }_{\tau _{k,2} \mathrm{th ~TA}}, \cdots ,\underbrace{\textbf{0}_{1 \times N_{\textrm{rf}} } }_{N_{\textrm{t}} \mathrm{th~ TA}}]^T \\ \end{array} \end{aligned}$$

Finally, according to the above design, after being modulated into one STBC codeword with both the CGI bits ($I_{\textrm{CGI}}$) and the mapping bits ($I_{\textrm{P}}$), the STBC codeword is conveyed over the corresponding channel states on two specified TAs with the AI bits ($I_{\textrm{AI}}$) during two time-slots by using the CI bits ($I^{1}_{\textrm{rf}}, I^{2}_{\textrm{rf}}$). Hence, the SE (: bits/s/Hz) of the STBC-SCM is obtained as

4 $$\begin{aligned} \eta = \frac{1}{2}\left( {\log _2 (M_1 M_2 ) + \log _2 4 + \left\lfloor {\log _2 C_{N_{\textrm{t}} }^\textrm{2} } \right\rfloor + 4n_{\textrm{rf}} } \right) . \end{aligned}$$

To further explain the design of the STBC-SCM, some examples of generating the MBM signal vector during two time-slots are provided in TABLE 1. Assuming that $N_{\textrm{t}}=4$, ${\Gamma }=\{ [1~0~1~0]^T,[1~0~0~1]^T,[0~1~1~0]^T,[0~1~0~1]^T\}$, $I^1_{\textrm{rf}}=\{I^{1, t_1}_{\textrm{rf}},I^{1, t_2}_{\textrm{rf}}\}$ and $I^2_{\textrm{rf}}=\{I^{2, t_1}_{\textrm{rf}},I^{2, t_2}_{\textrm{rf}}\}$. In TABLE 1, the $\textbf{0}$ denotes the zero vector with $1 \times 4$ dimensions.

**Table 1:** Generating the MBM vectors with $N_{\textrm{t}}=4$.

$I_{\textrm{AI }}, I^1_{\textrm{rf }},I^2_{\textrm{rf }}$	$N_{\textrm{t}}=4$, $n_{\textrm{rf}}=2$	
	$\textbf{X}$ of Eq. (1)	$\textbf{X}_{\textrm{M}}$ of Eq. (2)
{00}, {10,01},{01,11}	$$\left[ \begin{array}{l} x_1 ~~~~0~x_2 0 \\ - x_2^* ~0~x_1^* 0 \\ \end{array} \right] ^T$$	$$\left[ \begin{array}{l} {[0~0~x_1~0] } ~~~~\textbf{0} {[0~x_2~0~0] } \textbf{0} \\ { [0~- x_2^*~0~0 ] } \textbf{0} {[0~ 0~0~x_1^*] } \textbf{0} \\ \end{array} \right] ^T$$
{01}, {00,11},{10,11}	$$\left[ \begin{array}{l} x_1 ~~~~00~x_2 \\ -x_2^* ~0 0~x_1^* \\ \end{array} \right] ^T$$	$$\left[ \begin{array}{l} {[x_1~0~0~0 ] } ~~~~ \textbf{0} ~ \textbf{0} {[0~0~x_2~0] } \\ { [0 ~0~0~-x_2^*] } \textbf{0} ~ \textbf{0} {[0~0~0~x_1^*] } \\ \end{array} \right] ^T$$
{10}, {01,10},{00,10}	$$\left[ \begin{array}{l} 0~~~~~x_1 x_2 ~0 \\ 0~ - x_2^* x_1^*~ 0 \\ \end{array} \right] ^T$$	$$\left[ \begin{array}{l} \textbf{0} [0~x_1 ~0~0] ~~~~~ [x_2 ~0~0~0] \textbf{0} \\ \textbf{0} [0~0~-x_2^*~0] ~ [0~0~x_1^*~0] \textbf{0} \\ \end{array} \right] ^T$$
{11}, {11,00},{00,01}	$$\left[ \begin{array}{l} 0~~~~x_1 ~0~x_2 \\ 0 - x_2^* ~ 0~x_1^*\\ \end{array} \right] ^T$$	$$\left[ \begin{array}{l} \textbf{0}[0~0~0~x_1 ]~~~~\textbf{0}[x_2~ 0~0~0] \\ \textbf{0}[ - x_2^* ~0~0~0]~\textbf{0}[0~x_1^* ~0~0] \\ \end{array} \right] ^T$$

### Receiver

Based on the expression of (2) and (3), after the MBM vector ${ \tilde{\textbf{X}}}_{\textrm{M}}$ being transmitted over the specified fading channels of the channel matrix $\textbf{H}$ with the CI information during two time-slots, the received vector $\textbf{y} \in C^{N_{\textrm{r}} \times 2}$ is derived by

5 $$\begin{aligned} \begin{array}{l} \textbf{y} = \rho \cdot \textbf{H}{\tilde{\textbf{X}}}_{\textrm{M}} + \textbf{n} = \rho \cdot \left[ {\begin{array}{*{20}c} {\textbf{H}}{\tilde{\textbf{X}}}_{t_1 } & {\textbf{H}}{\tilde{\textbf{X}}}_{t_2 } \\ \end{array}} \right] + \textbf{n} \\ \end{array}, \end{aligned}$$

where the additive white Gaussian noise matrix $\textbf{n}\in C^{N_{\textrm{r }} \times 2}$ is independent of the transmit antenna configuration, with each entry being i.i.d. $CN(0,\sigma ^2 \textbf{I}_{N_{\textrm{r}} } )$. The channel matrix $\textbf{H} \in C^{N_{\textrm{r}}\times N_{\textrm{rf}} N_{\textrm{t}}}$ is denoted by

6 $$\begin{aligned} \begin{array}{l} \textbf{H} = [\textbf{H}_1 , \cdots ,\textbf{H}_{N_{\textrm{t}} } ] = [\underbrace{\textbf{h}_1^1 , \cdots ,\textbf{h}_{N_{r\textrm{f}} }^1 }_{1\mathrm{- st TA}}, \cdots ,\underbrace{\textbf{h}_1^{N_{\textrm{t}} } , \cdots ,\textbf{h}_{N_{r\textrm{f}} }^{N_{\textrm{t}} } }_{N_{\textrm{t}} \mathrm{- th TA}}] \\ \end{array}, \end{aligned}$$

whose each item obeys the Rayleigh fading with *CN*(0, 1). $\rho$ is a normalized factor calculated by $\rho \mathrm{= }{1 / {E_{\textrm{av}} }}$, $E_{\textrm{av}}$ denotes the average energy per STBC codeword in each time slot, and is obtained by the following calculation. Due to that four constellation groups: {$(\Upsilon ^\textrm{Q},\Upsilon ^\textrm{F} )$, $(\Upsilon ^\textrm{F},\Upsilon ^\textrm{Q} )$, $(\Upsilon ^\textrm{Q},\Upsilon ^\textrm{S} )$, $(\Upsilon ^\textrm{S},\Upsilon ^\textrm{Q} )$} with the specification of the CGI bits are used to be encoded into four different types of STBC codewords, e.g., $$\left[ {\begin{array}{*{20}c} {x^\textrm{Q} } & { - (x^\textrm{F} )^* } \\ {x^\textrm{F} } & {(x^\textrm{Q} )^* } \\ \end{array}} \right] ,\left[ {\begin{array}{*{20}c} {x^\textrm{F} } & { - (x^\textrm{Q} )^* } \\ {x^\textrm{Q} } & {(x^\textrm{F} )^* } \\ \end{array}} \right]$$, $$\left[ {\begin{array}{*{20}c} {x^\textrm{Q} } & { - (x^\textrm{S} )^* } \\ {x^\textrm{S} } & {(x^\textrm{Q} )^* } \\ \end{array}} \right] ,\left[ {\begin{array}{*{20}c} {x^\textrm{S} } & { - (x^\textrm{Q} )^* } \\ {x^\textrm{Q} } & {(x^\textrm{S} )^* } \\ \end{array}} \right]$$. According to the above four types of STBC codewords, it can be observed that they contain eight symbols ($x^\textrm{Q}$) drawn from $M_1$-QAM ($:\Upsilon ^\textrm{Q}$), four symbols ($x^\textrm{S}$) drawn from $M_2$-FSQAM ($:\Upsilon ^\textrm{F}$) and four symbols ($x^\textrm{S}$) drawn from $M_2$-SSQAM ($:\Upsilon ^\textrm{S}$) during two time-slots. Hence, the average energy $E_{\textrm{av}}$ per STBC codeword in each time slot can be calculated by

7 $$\begin{aligned} E_{\textrm{av}} = \frac{{4E_{\textrm{av}}^\textrm{Q} + 2E_{\textrm{av}}^{\textrm{SS}} + 2E_{\textrm{av}}^{\textrm{FS}} }}{\textrm{4}}, \end{aligned}$$

where $E_{\textrm{av}}^\textrm{Q}, E_{\textrm{av}}^{\textrm{SS}}, E_{\textrm{av}}^{\textrm{FS}}$ are the average energy per signal CPs in $M_1$-QAM, $M_2$-FSQAM, $M_2$-SSQAM, respectively.

At the receiver, the ML algorithm is employed for the STBC-SCM, which can be expressed mathematically as

8 $$\begin{aligned} \begin{array}{l} [{\hat{M}}_1 ,{\hat{M}}_2 ,{\hat{k}},{\hat{\alpha }} _1 ,{\hat{\alpha }} _2 ,{\hat{\beta }} _1 ,{\hat{\beta }} _2 ] = \arg \mathop {\min }\limits _{\textrm{par}} \left\| {\textbf{y} - \textbf{H} \cdot \rho \cdot { \tilde{\textbf{X}}}_{\textrm{M}} } \right\| ^2_F = \arg \mathop {\min }\limits _{\textrm{par} } \left\| {\left[ \begin{array}{l} \textbf{y}_{t_1 } \\ \textbf{y}_{t_2 } \\ \end{array} \right] - (\textbf{I}_2 \otimes \textbf{H}) \cdot \rho \cdot \left[ \begin{array}{l} { \tilde{\textbf{X}}}_{t_1 } \\ { \tilde{\textbf{X}}}_{t_2 } \\ \end{array} \right] } \right\| ^2_F \\ \end{array} \end{aligned}$$

where $\textrm{par}=\{M_1 ,M_2 ,k,\alpha _1 ,\alpha _2 ,\beta _1 ,\beta _2\}$, $\otimes$ denotes the operation of Kronecker product and $\textbf{y}_{t_1 }=\rho \cdot \textbf{H}{ \tilde{\textbf{X}}}_{t_1 }+\textbf{n}_{t_1}$ and $\textbf{y}_{t_2 }=\rho \cdot \textbf{H}{ \tilde{\textbf{X}}}_{t_2 }+\textbf{n}_{t_2}$. ${{\hat{M}}}_1,{{\hat{M}}}_2$ are respectively the estimates of the modulation orders $M_1,M_2$. ${\hat{k}}$ is the estimate of the AI vector number *k*, and ${\hat{\alpha }}_1 ,{\hat{\alpha }}_2$, ${\hat{\beta }}_1 ,{\hat{\beta }}_2$ are the estimates of the CI indexes for the specified $\alpha _1$- , $\alpha _2$-, $\beta _1$- ,$\beta _2$-th channel states, respectively. $\big \Vert \cdot \big \Vert ^2_F$ denotes the Frobenius norm.

## Performance analysis

### Squared MED

Although one STBC symbol needs two time-slots to be transmitted for achieving the transmit diversity, the squared MED between the spatial MBM vectors $\textbf{X}_{t_1}$/$\textbf{X}_{t_2}$ play an important role in reliability of communication in the process of transmission. In this paper, keeping the merit of transmit diversity, the CGI domain is developed for increasing the extra information. In other words, the squared MED between the MBM vectors are potentially improved at each time-slot. i.e., The enhancement for the squared MED of the STBC-SCM stems directly from: (i) Hybrid constellations increasing inter-vector distance (Table 2), (ii) Per-symbol CI allocation reducing channel-state collision probability. The squared MED for each time-slot is derived as

9 $$\begin{aligned} d_{{ \tilde{\textbf{X}}}_\mu }^2 = \mathop {\min }\limits _{{ \tilde{\textbf{X}}}_\mu \ne { \hat{{\tilde{\textbf{X}}}}}_\mu } \left\| {{ \tilde{\textbf{X}}}_\mu - { \hat{{\tilde{\textbf{X}}}}}_\mu } \right\| ^2 ,\mu \in \{ t_1 ,t_2 \}, \end{aligned}$$

which is analyzed in TABLE 2, here ($M_1, M_2$), (${\bar{M}}_1, {\bar{M}}_2$), ($M'_1, M'_2$), (${\bar{M}}'_1, {\bar{M}}'_2$) are respectively defined for the STBC-SCM, STCM-III, ST-QSM and QSM-MBM, where ${\bar{M}}_1, {\bar{M}}_2$, $M'_1, M'_2$, ${\bar{M}}'_1, {\bar{M}}'_2$ are the modulation orders of the QAM constellation, and $p_1$TX$p_2$b denotes the SE of $p_2$ bits/s/Hz in the scenario of $p_1$ TAs.

**Table 2:** Squared MED comparisons between the MBM vectors with the $t_1/t_2$-th time-slot.

Schemes	$N_{\textrm{t}}=2,~ n_{\textrm{rf}}=2$			
	2TX14b	2TX16b	2TX18b	2TX20b
STCM-III	$\frac{4}{40},(32,32)$	$\frac{4}{84},(64,64)$	$\frac{4}{164},(128,128)$	$\frac{4}{340},(256,256)$
ST-QSM ($N_{\textrm{t}}=8$)	$\frac{2}{12},(8,8)$	$\frac{2}{20},(16,16)$	$\frac{2}{40},(32,32)$	$\frac{2}{84},(64,64)$
STBC-SCM	$\frac{4}{8},(4,4)$	$\frac{4}{16},(16,4)$	$\frac{4}{32},(32,8)$	$\frac{4}{67},(64,16)$
Schemes	$N_{\textrm{t}}=4$, $n_{\textrm{rf}}=2$		$N_{\textrm{t}}=4$, $n_{\textrm{rf}}=3$	
	4TX18b	4TX20b	4TX20b	4TX22b
ST-QSM ($N_{\textrm{t}}=\{16,32\}$)	$\frac{2}{12},(8,8)$	$\frac{2}{20},(16,16)$	$\frac{2}{4},(4,4)$	$\frac{2}{12},(8,8)$
QSM-MBM	$\frac{2}{12},(8,8)$	$\frac{2}{20},(16,16)$	$\frac{2}{12},(8,8)$	$\frac{2}{20},(16,16)$
STBC-SCM	$\frac{4}{16},(16,4)$	$\frac{4}{32},(32,8)$	$\frac{4}{8},(4,4)$	$\frac{4}{16},(16,4)$

Note that, in the ST-QSM system with ($M'_1, M'_2$) in TABLE 2, $N_{\textrm{t}}=8,~16,~32$ number of TAs are configured for these three scenarios of $\{N_{\textrm{t}}=2,~ n_{\textrm{rf}}=2\}$, $\{N_{\textrm{t}}=4,~ n_{\textrm{rf}}=2\}$, $\{N_{\textrm{t}}=4,~ n_{\textrm{rf}}=3\}$, respectively.

### Detection complexity analysis

The ML detector in Eq. (7) requires exhaustive search over all possible combinations of CGI, AI, CI, and symbol constellations. Due to the employment of four constellation groups: {$(\Upsilon ^\textrm{Q},\Upsilon ^\textrm{F} )$, $(\Upsilon ^\textrm{F},\Upsilon ^\textrm{Q} )$, $(\Upsilon ^\textrm{Q},\Upsilon ^\textrm{S} )$, $(\Upsilon ^\textrm{S},\Upsilon ^\textrm{Q} )$}, four distinct STBC codeword types are genrated: $$\left[ {\begin{array}{*{20}c} {x^\textrm{Q} } & { - (x^\textrm{F} )^* } \\ {x^\textrm{F} } & {(x^\textrm{Q} )^* } \\ \end{array}} \right] ,\left[ {\begin{array}{*{20}c} {x^\textrm{F} } & { - (x^\textrm{Q} )^* } \\ {x^\textrm{Q} } & {(x^\textrm{F} )^* } \\ \end{array}} \right]$$, $$\left[ {\begin{array}{*{20}c} {x^\textrm{Q} } & { - (x^\textrm{S} )^* } \\ {x^\textrm{S} } & {(x^\textrm{Q} )^* } \\ \end{array}} \right] ,\left[ {\begin{array}{*{20}c} {x^\textrm{S} } & { - (x^\textrm{Q} )^* } \\ {x^\textrm{Q} } & {(x^\textrm{S} )^* } \\ \end{array}} \right]$$. The detection complexity is averaged across these codeword types. For the operation of $\textbf{H} \cdot { \tilde{\textbf{X}}}_{\textrm{M}}$, $12N_{\textrm{r}}$, $12N_{\textrm{r}}$, $16N_{\textrm{r}}$, $16N_{\textrm{r}}$ real complexities are required for four STBC codeword types, respectively. Additionally, calculating the operation of $\left\| \cdot \right\| ^2$ needs $4N_{\textrm{r}}$ real complexities for each type of STBC codeword. Thus, the average complexity is $18N_{\textrm{r}} \cdot \eta$ real operations, where $\eta$ is the SE. In contrast, the STCM-III and the QSM-MBM systems require $20N_{\textrm{r}} \cdot \eta$ real complexities.Consequently, compared with the STCM-III and the QSM-MBM systems, the STBC-SCM reduces the real complexity by $2N_{\textrm{r}} \cdot \eta$. Next, Future work will explore low-complexity alternatives (e.g., modified sphere decoding) for practical implementation.

### Average BEP analysis

In what follows, the average BEP upper bound of the STBC-SCM is analyzed.

Assume that ${ \tilde{\textbf{X}}_{\textrm{M}}}$ is transmitted and mistakenly detected as ${{\hat{\tilde{\textbf{X}}}}_{\textrm{M}}}$. Based on the ML detection in Eq. (8) and Ref.^24^, the well-known conditional pairwise error probability (PEP) can be obtained as

10 $$\begin{aligned} P({ \tilde{ \textbf{X}}}_{\textrm{M}} \rightarrow { \hat{{\tilde{\textbf{X}}}}}_{\textrm{M}} \left| \textbf{H} \right. ) = P(\left\| {\textbf{y} - \rho \cdot { H{{\tilde{\textbf{X}}}}}_{\textrm{M}} } \right\| _F^2> \left\| {\textbf{y} - \rho \cdot {H\hat{{\tilde{\textbf{X}}}}}_{\textrm{M}} } \right\| _F^2 \left| \textbf{H} \right. ) = P(Z > \left\| {\rho \cdot \textbf{H}({\hat{{\tilde{\textbf{X}}}}}_{\textrm{M}} - {{{\tilde{\textbf{X}}}}}_{\textrm{M}} )} \right\| _F^2 ), \end{aligned}$$

where $\mu = \textrm{Tr}\left\{ {\textbf{n} \cdot [\rho \textbf{H}({\tilde{\textbf{X}}}_{\textrm{M}} - {\hat{{\tilde{\textbf{X}}}}}_{\textrm{M}} )]^H + [\rho \textbf{H}({ \tilde{ \textbf{X}}}_{\textrm{M}} - {\hat{{\tilde{\textbf{X}}}}}_{\textrm{M}} )] \cdot \textbf{n}^H } \right\}$ is a zero mean Gaussian random variable with variance $2\sigma ^2 \cdot \rho ^{ 2} \left\| {\textbf{H}({ \tilde{\textbf{X}}}_{\textrm{M}} - {\hat{{\tilde{\textbf{X}}}}}_{\textrm{M}} )} \right\| _F^2$, Tr$\{\cdot \}$ is the trace of a matrix. Thus, using the *Q*(*x*) function, the Eq. (10) can be rewritten as

11 $$\begin{aligned} P({ \tilde{\textbf{X}}}_{\textrm{M}} \rightarrow {\hat{{\tilde{\textbf{X}}}}}_{\textrm{M}} |\textbf{H}) = Q\left( {\sqrt{\frac{{\left\| {\textbf{H}({ \tilde{\textbf{X}}}_{\textrm{M}} - {\hat{{\tilde{\textbf{X}}}}}_{\textrm{M}} )} \right\| _F^2 }}{{2\sigma ^2 \rho ^{ - 2} }}} } \right) = Q\left( {\sqrt{\frac{{\mathrm{Tr[} \textbf{H} \cdot \Delta \cdot \textbf{H}^H \mathrm{]}}}{{2\sigma ^2 \rho ^{ - 2} }}} } \right) \end{aligned}$$

where $\Delta = ({ \tilde{\textbf{X}}}_{\textrm{M}} - {\hat{{\tilde{\textbf{X}}}}}_{\textrm{M}} )({{{\tilde{\textbf{X}}}}}_{\textrm{M}} - {\hat{{\tilde{\textbf{X}}}}}_{\textrm{M}} )^H$ is a metric on the STBC codeword.

Then, according to {Ref.^24^, P46-52} and calculating the expected value of (11) with the items of the matrix $\textbf{H}$, the upper bound on *Q* function $Q(x) \le \frac{1}{2}e^{{{ - x^2 }/ 2}}$ is employed, since $E\left\{ {\textbf{H} \cdot \textbf{H}^H } \right\} = N_{\textrm{r}} I_{N_{\textrm{t}} }$ the upper bound of the average PEP can be derived as

12 $$\begin{aligned} P({ \tilde{\textbf{X}}}_{\textrm{M}} \rightarrow {\hat{{\tilde{\textbf{X}}}}}_{\textrm{M}} ) \le \frac{1}{2}\exp \left( { - \frac{{N_{\textrm{r}} \textrm{Tr}\left\{ \Delta \right\} }}{{4\sigma ^2 \rho ^{ - 2} }}} \right) . \end{aligned}$$

Consequently, with a closed-form solution in Ref.^24^, the upper bound on the average BEP can be given by

13 $$\begin{aligned} P_b \le \frac{1}{{2^B }}\sum \limits _{{{{\tilde{\textbf{X}}}}}_{\textrm{M}} } {\sum \limits _{{\hat{{\tilde{\textbf{X}}}}}_{\textrm{M}} \ne {{\hat{\textbf{X}}}}_{\textrm{M}} } {\frac{{P\left( {{{{\tilde{\textbf{X}}}}}_{\textrm{M}} \rightarrow {\hat{{\tilde{\textbf{X}}}}}_{\textrm{M}} } \right) \cdot e\left( {{{{\tilde{\textbf{X}}}}}_{\textrm{M}} ,{\hat{{\tilde{\textbf{X}}}}}_{\textrm{M}} } \right) }}{B}} }, \end{aligned}$$

where $e(\tilde{\textbf{X}}_{\textrm{M}}\rightarrow { \hat{\tilde{\textbf{X}}}_{\textrm{M}}})$ denotes the total number of erroneous bits between $\textbf{X}_{\textrm{M}}$ and $\hat{\tilde{\textbf{X}}}_{\textrm{M}}$.

## Simulation and numerical results

The BERs of the STBC-SCM compared with the classic MBM variants with $n_{\textrm{rf}}=\{2,3\}$ mirrors are presented for the same TAs of $N_{\textrm{t}}=\{2,4\}$. Simulation results are with the ML detector and the known channel state information at the receiver, $p_1$TX$p_2$b denotes the SE of $p_2$ bits/s/Hz with $p_1$ TAs in two time-slots. In order to observe intuitively the comparison results, we can define that the STBC-SCM with ($M_1,M_2,n_{\textrm{rf}}$), the STCM-III^3^ with (${\bar{M}}_1, {\bar{M}}_2,n_{\textrm{rf}}$), the ST-QSM^13^ with ($M'_1, M'_2$) and the QSM-MBM^22^ with (${\bar{M}}'_1, {\bar{M}}'_2,n_{\textrm{rf}}$).

**Figure 2:**
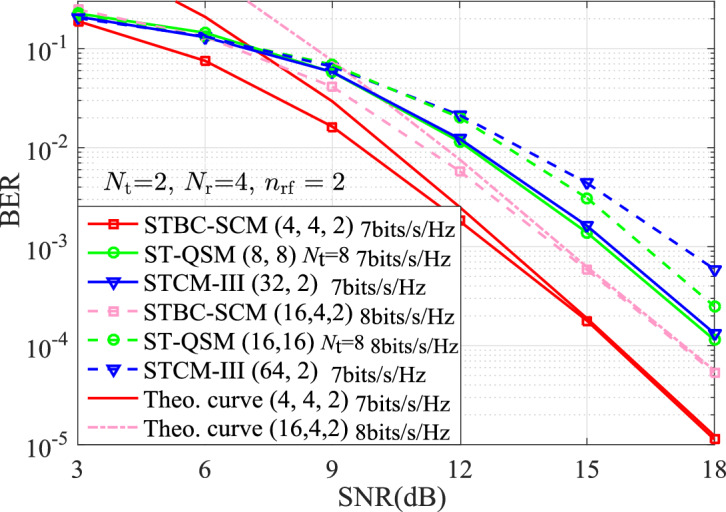
BER comparison between the STBC-SCM and the ST-QSM, the STCM-III at both the solid-line curves for 2TX14b and the dashed curves for 2TX16b when $N_{\textrm{r}}=4,~n_{\mathrm{{rf}}}=2$, $\eta = \{7,8 \}$ bits/s/Hz.

In Fig. 2, at 2TX14b and 2TX16b with $N_{\textrm{r}}=4$, it can be observed that the effectiveness of the STBC-SCM has been validated due to that the theoretical and simulation results are in agreement in the high signal noise ratio (SNR) region. In addition, simulation results for the STBC-SCM are compared with the STCM-III with 2TX14b and 2TX16b, the ST-QSM with 8TX14b and 8TX16b. It is thus clear that, the STBC-SCM with the absence of AI bits achieves the better BER performance. For instance, about 2.7 dB SNR gains over the STCM-III with 2TX14b and 2.2 dB SNR gains over the ST-QSM with 8TX14b at the BER value of $10^{-3}$ are obtained due to the bigger squared MED reported in TABLE 2.

**Figure 3:**
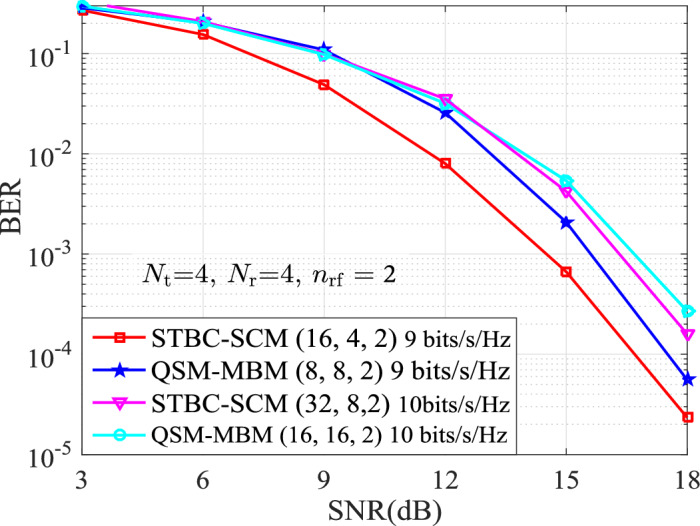
BER Performances for both the STBC-SCM and QSM-MBM at 4TX18b and 4TX20b with $n_{\textrm{rf}}=2$, $\eta = \{9,10 \}$ bits/s/Hz.

**Figure 4:**
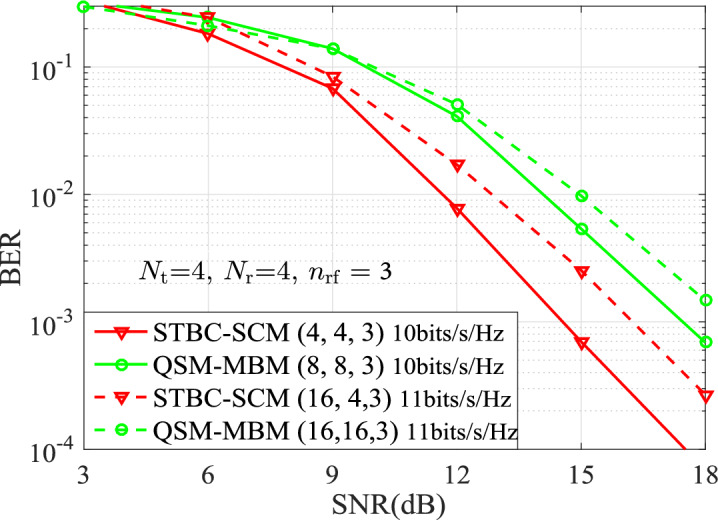
BER Performances for both the STBC-SCM and QSM-MBM at 4TX20b and 4TX22b with $n_{\textrm{rf}}=3$, $\eta = \{10,11 \}$ bits/s/Hz.

In order to further outstand the advantage of the STBC-SCM, Fig. 3 and Fig. 4 present the BER curves of the STBC-SCM and compared with the QSM-MBM at 4TX18b, 4TX20b and 4TX22b with $n_{\textrm{rf}}=\{2,3\}$. In Fig. 3 with $n_{\textrm{rf}}=2$, it can be observed that, the STBC-SCM lowers the BER values at 4TX18b and 4TX20b, such as 1 dB SNR gain over the QSM-MBM with (8, 8, 2) at the BER value of $10^{-3}$. Furthermore, according to the squared MED analyzed in TABLE 2, the STBC-SCM depicted in Fig. 4 with $n_{\textrm{rf}}=3$ achieves the significant BER performance over the QSM-MBM with both (8, 8, 3) and (16, 16, 3) at the same SEs of 4TX20b and 4TX22b. For instance, compared with the QSM-MBM with (8, 8, 3) at 4TX20b, approximately 3 dB SNR gains are achieved by the STBC-SCM at the BER value of $10^{-3}$.

**Figure 5:**
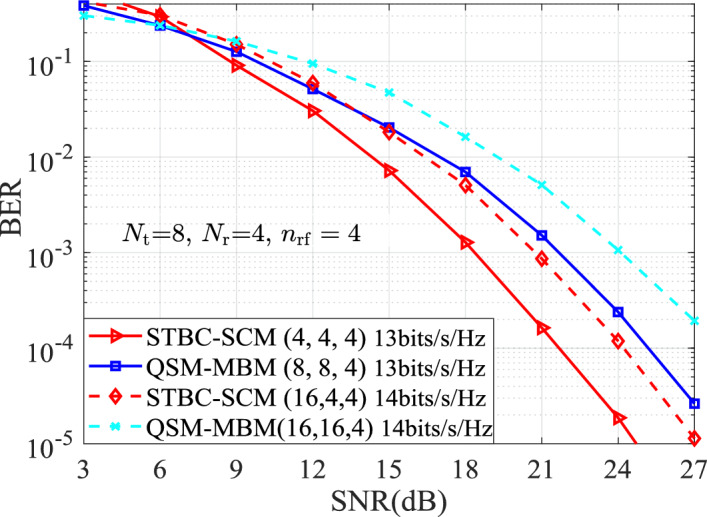
BER Performances for both the STBC-SCM and QSM-MBM at 8TX26b and 8TX28b with $n_{\textrm{rf}}=4$, $\eta = \{13,14 \}$ bits/s/Hz.

Fig. 5 presents the BER performance of the proposed STBC-SCM system at the SEs of $\{13,14\}$ bits/s/Hz, $n_{\text {rf}} = 4$ RF mirrors per transmit antenna (yielding 16 distinct channel states per TA). The results are compared against the QSM-MBM at the same configurations. It can be observed from Fig. 5 that, at least 2.5 dB SNR gains are achieved for the proposed STBC-SCM over the the QSM-MBM at the BER value of $10^{-3}$. Therefore, with increasing of RF mirrors on each TA, the proposed STBC-SCM also achieve significant SNR gain over the QSM-MBM.

## Conclusion

In this paper, we have presented the STBC-SCM system, which can further enhance the SE by developing more extra information including the CGI bits and the CI bits. Firstly, four groups with mixing two signal constellations are designed for the selection of two CGI bits. Furthermore, the resulted STBC codeword is modulated by the specified AI vector with the AI bits on two specified active TAs. Then, four symbols from the STBC codeword on two active TAs during two time-slots are transmitted over the specified channel states with four independent parts of CI bits. The squared MED for each time-slot and Average BEP were analyzed. Also, numerical results is shown that the STBC-SCM outperform the existing classic MBM schemes such as STCM, QSM-MBM in terms of the error performance. Finally, for the STBC system, the joint design of constellations group and channel modulation aims to exploit the additional information from the indexes of constellations group and channel states, which is worth further research to improve the throughput and error performance. Furthermore, investigating the impact of channel correlation on the performance of the CI bits, which affects the discernibility of different channel states, remains an important topic for future research.

## Data Availability

The datasets used and/or analysed during the current study available from the corresponding author on reasonable request.
